# Soluble amyloid beta oligomers block the learning-induced increase in hippocampal sharp wave-ripple rate and impair spatial memory formation

**DOI:** 10.1038/srep22728

**Published:** 2016-03-07

**Authors:** Olivier Nicole, Senka Hadzibegovic, Judyta Gajda, Bruno Bontempi, Tiaza Bem, Pierre Meyrand

**Affiliations:** 1 Institut des Maladies Neurodégénératives, Université de Bordeaux, UMR 5293, 33000 Bordeaux, France; 2 CNRS, Institut des Maladies Neurodégénératives, UMR 5293, 33000 Bordeaux, France; 3 Nalecz Institute of Biocybernetics and Biomedical Engineering, Polish Academy of Sciences, 02-109, Warsaw, Poland

## Abstract

Post-learning hippocampal sharp wave-ripples (SWRs) generated during slow wave sleep are thought to play a crucial role in memory formation. While in Alzheimer’s disease, abnormal hippocampal oscillations have been reported, the functional contribution of SWRs to the typically observed spatial memory impairments remains unclear. These impairments have been related to degenerative synaptic changes produced by soluble amyloid beta oligomers (Aβos) which, surprisingly, seem to spare the SWR dynamics during routine behavior. To unravel a potential effect of Aβos on SWRs in cognitively-challenged animals, we submitted vehicle- and Aβo-injected mice to spatial recognition memory testing. While capable of forming short-term recognition memory, Aβ mice exhibited faster forgetting, suggesting successful encoding but an inability to adequately stabilize and/or retrieve previously acquired information. Without prior cognitive requirements, similar properties of SWRs were observed in both groups. In contrast, when cognitively challenged, the post-encoding and -recognition peaks in SWR occurrence observed in controls were abolished in Aβ mice, indicating impaired hippocampal processing of spatial information. These results point to a crucial involvement of SWRs in spatial memory formation and identify the Aβ-induced impairment in SWRs dynamics as a disruptive mechanism responsible for the spatial memory deficits associated with Alzheimer’s disease.

Information processing and memory formation in rodents have been reported to be accompanied by an array of hippocampal field potential oscillations that are important functionally. For instance, theta oscillations occur during active behavior and rapid eye movement (REM) sleep and have been suggested to provide the temporal frame for the encoding of information^1^. Gamma oscillations triggered during exploratory behavior are thought to be involved in memory acquisition^2^ and their synchronization contributes to successful execution of working memory^3^. During slow wave sleep (SWS) that follows learning, hippocampal circuits consistently increase the occurrence rates of sharp wave-ripples (SWRs) which typically recur at 0.4 to 1 Hz^4^,^5^. Importantly, upon occurrence of SWRs, ensembles of hippocampal place cells can replay in faster timescales their sequential activity triggered during a previous learning episode, suggesting an essential role for SWRs in driving memory consolidation processes and subsequent long-term stabilization of newly acquired spatial memory traces^6^. When such SWRs are experimentally disrupted, it causes memory deficits in hippocampus-dependent memory tasks^7^, further suggesting that abnormal hippocampal rhythmic activity can interfere with hippocampal information processing, a dysfunctional pattern also observed in pathological conditions such as Alzheimer’s disease^8^ (AD).

The cognitive impairments associated with AD are related to degenerative synaptic changes produced by the presence of soluble amyloid beta proteins (Aβs) in vulnerable brain regions such as the hippocampus considered to be critical for spatial learning and declarative memory^9^. There is increasing evidence that early oligomeric forms of Aβs, rather than late fibrillar conformations, interfere with neuronal network functional properties and are responsible for cognitive dysfunctions in AD patients^10^ as well as in transgenic mouse models of this disease^11^. It has been found that Aβ oligomers (Aβos) differentially affects hippocampal network activities, reducing theta and gamma oscillations *in vitro*^12^ while surprisingly sparing SWRs^13^. Close examination reveals that such a lack of effect may be the consequence of recording hippocampal activity either in cell cultures or in animals remaining in their home cage, a basal condition which may hinder an effect of Aβos on SWRs otherwise detectable in cognitively-challenged animals. Here, we sought to unravel the action of Aβos on neuronal populations involved in the generation of SWRs in mice undergoing encoding and consolidation of spatial information. To this end, we submitted mice to spatial recognition memory testing in a modified version of the Y-maze discrimination task tailored to maximizing spatial cognitive demand. After confirming the hippocampal-dependency of this task, we established its ability to detect spatial memory impairments after intracerebroventricular infusion of Aβos. We then determined the signature of this Aβo treatment on hippocampal SWRs in mice without cognitive requirements or while undergoing a single spatial discrimination session.

## Results

### Aβos impair the formation of spatial recognition memory

**Figure 1:**
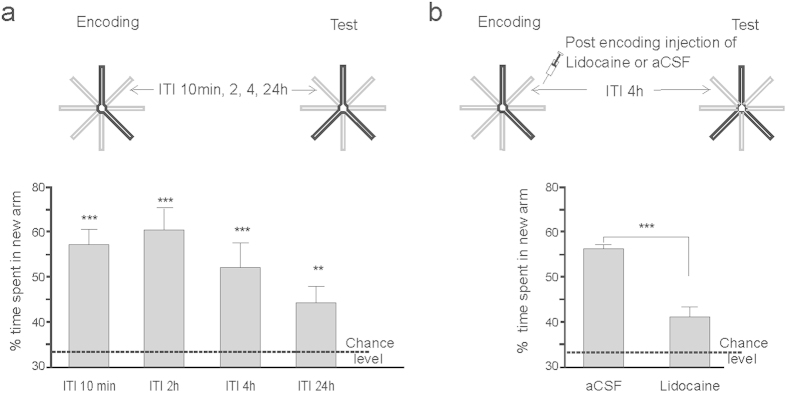
Spatial recognition memory testing in a modified version of the Y-maze discrimination task. (**a**) Recognition of the novel arm is long-lasting as shown by its persistence over increasing ITIs between encoding and recognition phases of the testing procedure in the 8-arm radial maze setup (n = 15 for ITI 10 min and 4 h, n = 14 for ITI 24 h and n = 11 for ITI 2 h, **p < 0.01; ***p < 0.001 versus chance level, t-tests (**b**) Silencing of hippocampal activity with lidocaine infused after encoding impairs recognition memory probed 4 hours later compared to mice injected with vehicle (aCSF)(n = 9/group, t_16_ = 5.85, ***p < 0.0001).

To characterize the effects of Aβos on spatial recognition memory and training-induced hippocampal SWR, we used a modified version of the Y-maze two-trial arm discrimination task conducted in an 8-arm radial maze apparatus and designed to increasing spatial cognitive demand (Fig. 1a). As expected, after a single encoding phase of 10 min with only two arms accessible, a short inter-trial interval (ITI) of 10 min resulted in a robust preference for the unexplored (previously closed) arm during the test phase (Fig. 1a). Interestingly, increasing the ITI from 10 min to 24 hours revealed spatial recognition memory performance that was still above chance within this extensive time window (Fig. 1a). Importantly, bilateral region-specific inactivation of the hippocampus with the sodium channel blocker lidocaine infused immediately after encoding impaired recognition memory probed 4 hours later (n = 9/group, t_16_ = 5.85, p < 0.0001), thus confirming the supportive role of the hippocampus in the formation and expression of recognition memory (Fig. 1b).

**Figure 2:**
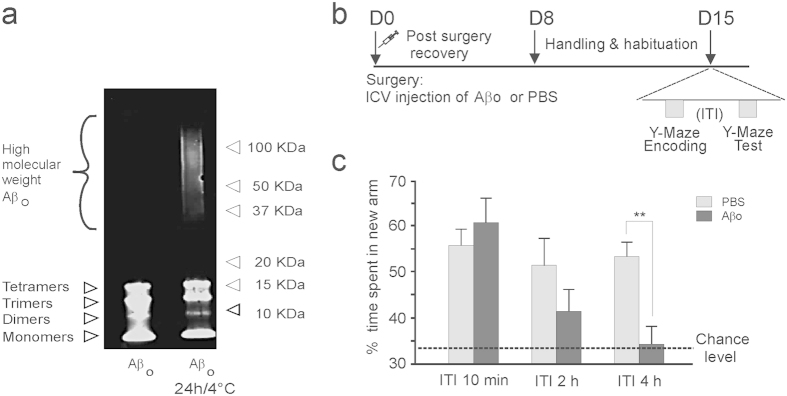
Aβos impair spatial recognition memory in a time-dependent manner. (**a**) Immunoblot analysis of the Aβo solution injected intracerebroventrically showing the aggregation states of Aβos before and after 24 h of incubation at 4 °C. Monomers, dimers, trimers and tetramers were present in the freshly prepared solution. High molecular weight of Aβ_(1–42)_ assemblies ranging from 30 to 100 kDa were also detected after 24 h of incubation. (**b**) Experimental design is shown. (**c**) While recognition memory performance in Aβ mice was similar to PBS-controls after 10 min (n = 6), it started to decrease as the ITI between encoding and test increased from 2 (n = 8–9) to 4 h. At the longer ITI, Aβ mice (n = 11) were severely impaired compared to PBS-control mice (n = 12), indicating faster forgetting (treatment x delay interaction F_2,39_ = 3.48, p < 0.05, **p < 0.01 versus PBS-controls).

We next sought to unravel the impact of Aβos on memory performance. We used a standardized assay to generate oligomers from synthetic Aβ peptides. As previously reported^14^, Western blot analysis of Aβo preparation by using the monoclonal 6E10 antibody directed against the human β-amyloid peptide revealed the presence of Aβ_(1–42)_ monomers, dimers, trimers and tetramers under phosphate buffered saline (PBS) conditions (Fig. 2a). Larger oligomeric assemblies ranging from 30 to 100 kDa were also detected after incubation for 24 h at 4 °C. Smearing observed for larger oligomeric assemblies can possibly indicate interconversion between these assemblies during electrophoresis (Fig. 2a). Because various soluble Aβ oligomer species have been reported to induce cognitive deficits, including dimers, trimers, dodecamers, and larger soluble Aβ oligomers with molecular weights of 90 to 650 kDa (20 to 150 mers)^15^, we chose to inject an Aβo mixture incubated 24 h at 4 °C in which most of these species can be found. Fifteen days following a single intracerebroventricular injection of Aβos or vehicle (PBS), we examined recognition memory performance 10 minutes, 2 hours or 4 hours after encoding (Fig. 2b). Following the shorter retention delay, both PBS- and Aβo-injected mice spent more time in the novel arm compared to the familiar (previously visited) ones (Fig. 2c; n = 6/group). In contrast, when the retention delay between the encoding and the test phases increased, Aβo-injected mice exhibited poorer performance compared to controls. Although not significant, impairment started to emerge at the 2 h time-point. (Figure 2c, n = 8–9/per group). At the 4 h delay, Aβo-injected mice failed to discriminate the novel arm (Fig. 2c, n = 11–12 per group). They were severely impaired and performed at chance while vehicle-injected mice were still successful and exhibited a performance level similar to that observed after the short retention delay (Fig. 2c). This delay-dependent Aβo impairment was memory-specific as there was no confounding effect of the Aβ treatment on the total exploration time of arms of the maze during either the encoding phase (Aβ mice: 222.49 sec ± 29.36; PBS mice: 218.09 sec ± 23.25, t_21_ = 0.12, NS, n = 11–12) or the test phase (Aβ mice: 105.06 sec ± 23.24; PBS mice: 131.36 sec ± 26.74, t_21_ = 0.74, NS). Likewise, there was no preference for a particular arm (arm preference, two-way ANOVA, F_(1,42)_ = 0.0029, NS) and no effect of treatment on arm preference (arm preference × treatment interaction, F_(1,42)_ = 0.2415, NS) during the encoding phase (open arm 1: Aβ mice: 108.21 sec ± 13.69; PBS mice 112.82 sec ± 13.34; open arm 2: Aβ mice: 114.27 sec ± 16.6; PBS mice: 105.27 sec ± 11.69, n = 11–12). Collectively, these findings indicate that Aβo-injected mice were capable of processing visuo-spatial information and forming short-term recognition memory. However, when the retention delay was extended, they exhibited accelerated forgetting, a memory profile also observed in transgenic mouse models of AD^16^.

Aβos were injected intracerebroventrically to avoid hippocampal damage induced by the injection cannula that could have interfered with subsequent electrophysiological recordings in the hippocampus. To verify that Aβos invaded the hippocampus and were still present at the time of behavioral and electrophysiological assessments 15 days post-injection, we conducted experiments in which we measured the concentrations of the Aβ_(1–42)_ peptide in the hippocampus 1 day and 15 days following intracerebroventricular injection. Aβ_(1–42)_ peptides were detectable after 15 days. However, as could be expected, hippocampal Aβ_(1–42)_ peptide concentration was lower after 15 days (104.95 ± 41.6 pg/g of total proteins; n = 4) compared to 1 day (661,37 ± 243.67 pg/g of total proteins; n = 4), a decrease likely resulting from cerebral clearance. Altogether, these results indicate that the Aβo-induced behavioral and electrophysiological (see below) changes that we observed are related, at least in part, to the hippocampal amyloid pathology.

### Memory-induced hippocampal SWRs activity patterns are impaired by Aβos

**Figure 3:**
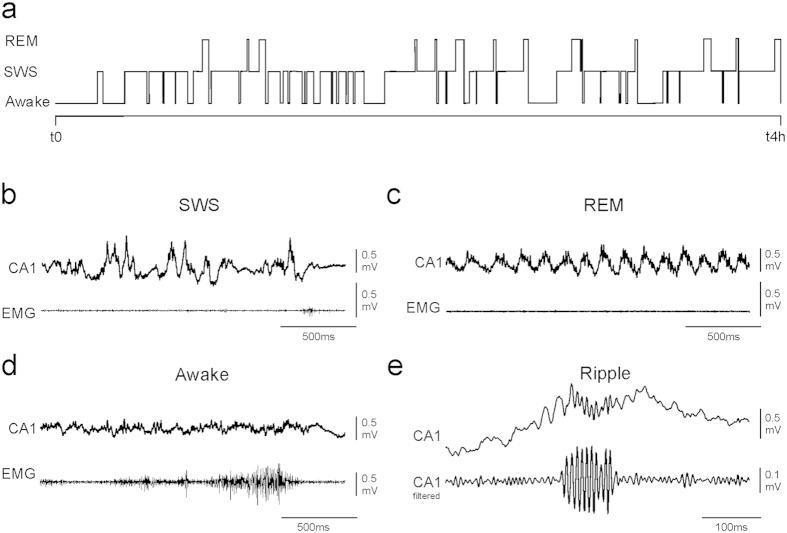
Representative examples of hippocampal LFP and EMG during different sleep and awake states. (**a**) Typical alternation in REM/SWS/awake over the 4 h time course separating encoding and recognition testing while the mouse remained in its home cage (see Fig. 5 for experimental paradigm). (**b**–**d**) Representative examples of LFP from the hippocampal CA1 region (CA1) and EMG during SWS (**b**), REM (**c**) and awake states (**d**). (**e**) Representative recordings of SWRs in the CA1 region of the hippocampus. EMG: extracellular recordings from neck muscles; CA1: LFP and filtered LFP recorded from hippocampal pyramidal cell layers.

**Figure 4:**
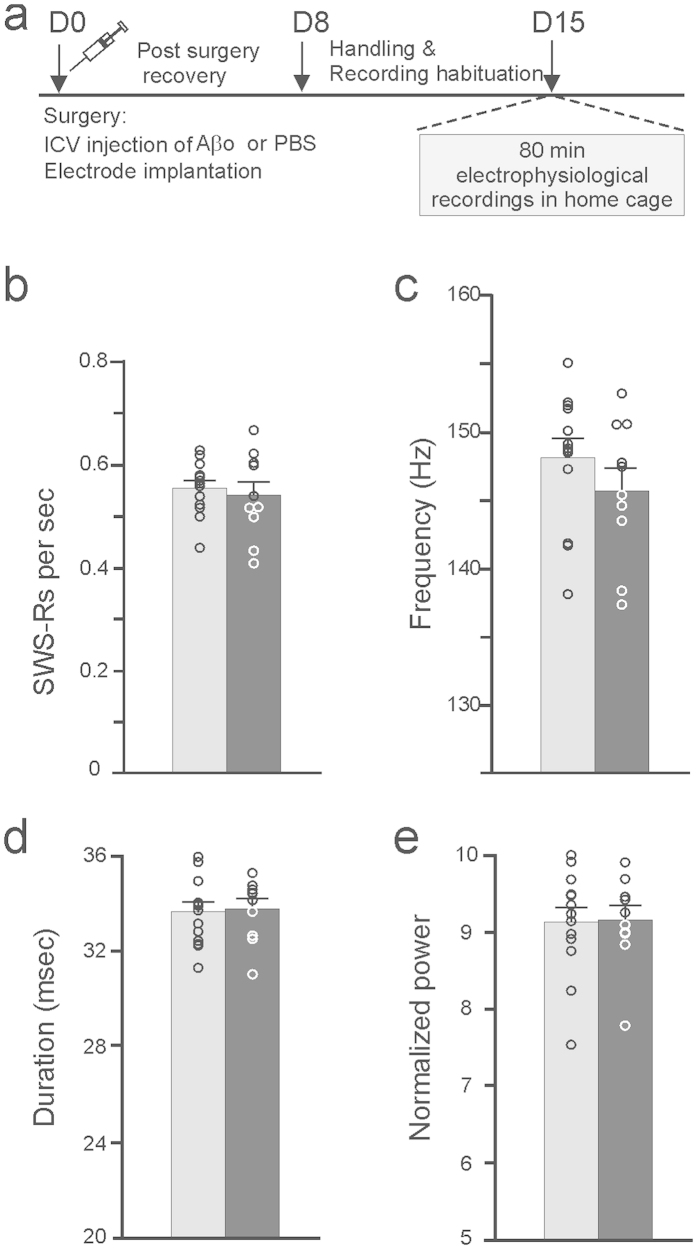
Characteristics of SWRs generated during baseline resting state in PBS-controls (gray bars, n = 13) and Aβo-injected (black bars, n = 10) mice. (**a**) Experimental design is shown. Occurrence rate (**b**), frequency (**c**), duration (**d**) and normalized power (**e**) of SWS-Rs were not affected by the Aβ treatment (p > 0.2 for all comparisons, t-test).

The memory profile of Aβo-injected mice points to an inability to form a stable memory over time and is suggestive of impaired consolidation processes during which SWRs are thought to play a privileged role^6^. To examine the effects of Aβos on the dynamics of SWRs, we recorded the extracellular field potential activity in the CA1 region which enabled us to pinpoint and characterize different sleep/awake stages triggered in our recognition memory paradigm. These hippocampal recordings were performed 15 days after intracerebroventricular injections of Aβos. A typical example of a SWS/REM/awake alternation as well as corresponding electromyogram (EMG) and local field potential (LFP) patterns for each of these states are illustrated in Fig. 3a–e. We focused our analysis only on SWRs occurring during the SWS bouts. When analyzing the characteristics of SWRs (baseline occurrence rate, frequency, duration and normalized power) during slow-wave sleep periods taking place when animals remained for 80 min in their home cage without behavioral challenge (Fig. 4a), we found that the overall SWR properties were left unaffected by the Aβo treatment (Fig. 4b–e). The occurrence rate, frequency, duration and normalized power of SWRs were very similar between PBS- and Aβo-injected groups (NS for all comparisons, t-test, n = 10–13). This finding is in agreement with previous observations showing no alteration of SWR properties by Aβo^13^. In sharp contrast, cognitively-challenged Aβ mice exhibited impaired SWR patterns compared to PBS-control mice (see below).

**Figure 5:**
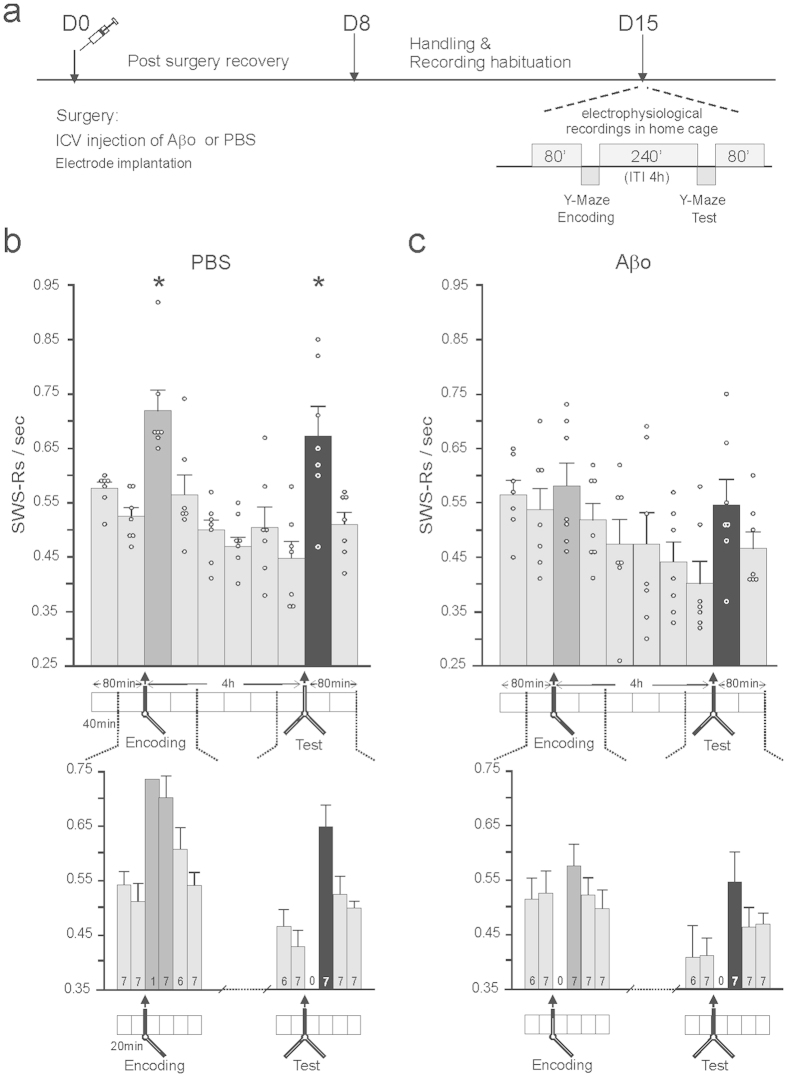
Time course of SWRs occurrence rate over 40 min time bin prior and after the encoding and test phases of the spatial recognition memory procedure in vehicle- and Aβo-injected mice. (**a**) Experimental design is shown. (**b**,**c**) Encoding- and recognition-induced peaks (depicted by dark gray and black bars, respectively) in SWR occurrence rates observed in PBS-controls (**b)**, upper panel, *p < 0.05 versus other measurements, Bonferroni t-test, n = 7) were abolished in Aβo-injected mice (**c**), upper panel, NS versus all other measurements, Bonferroni t-test, n = 7). A similar pattern of effects of Aβos on SWRs was observed over shorter time bins of 20 min (lower panels). Note that for the first post-encoding and post-test 20 min bins, animals generally did not express SWS episodes, preventing the assessment of SWRs associated with SWS (the first SWS episodes occurred at 23.72 ± 2.23 min and 23.43 ± 1.61 min in vehicle- and Aβo-injected mice, respectively).

To examine the effects of Aβos on SWRs occurrence as a function of the cognitive demand, we recorded the extracellular field potential activity in the CA1 region of the hippocampus over 10 time intervals of 40 minutes distributed as follows: baseline activity prior to memory encoding (2 intervals), encoding-induced activity (6 intervals) and testing-induced activity (2 intervals) (Fig. 5a). This segmented time course enabled us to pinpoint and characterize the dynamics of SWRs occurring in resting conditions and at different stages of spatial memory processing, namely encoding, consolidation and recognition. Since the memory deficit in Aβo-injected mice was significant at 4 h post-encoding, we only kept this time-point for our electrophysiological recordings. It must be noted that the occurrence rate and duration of SWS episodes over the time course of the experiment were similar in the two groups (occurrence rate: Aβ mice 7.1 per hour ± 0.61; PBS mice, 8.01 per hour ± 0.73; duration: Aβ mice, 5.36 min ± 0.5; PBS mice, 4.68 min ± 0.49; F < 1, NS for all comparisons, n = 7/per group). REM episodes were also similar in both groups (occurrence rate: Aβ mice 3.89 per hour ± 0.14, PBS mice 3.75 per hour ± 0.19; duration: Aβ mice 1.20 min ± 0.15; PBS mice 1.23 min ± 0.15; F < 1, NS). Also, the amount of SWS per 40 min bin was similar in both groups and ranged from 22.32 min ± 3.53 to 30.41 min ± 1.5 in PBS and from 23.15 min ± 3.56 to 33.46 min ± 1.02 in Aβo-injected animals with the exception of the post-encoding and post-test periods (bins 3 and 9, in which the first 20 min were usually occupied by awake state). During these specific time bins the amount of SWS ranged from 12.79 min ± 1.76 to 12.97 min ± 1.85 in PBS and from 12.24 min ± 2.05 to 14.22 min ± 1.95 in Aβo-injected group.

Interestingly, during the course of this experiment, two peaks of hippocampal SWRs occurrence were clearly apparent in PBS-control mice, one triggered upon exploration of the two available arms of the 8-arm radial maze, the other occurring upon the recognition phase of the testing procedure during which mice successfully identified the presence of the new open arm (see stars, upper panel, Fig. 5b). Indeed, ANOVA revealed a significant main effect of “time bins” in the PBS group (F_6,9_ = 16.16, p < 0.0001) which was due to an increase of post-learning (p < 0.05 versus all other measurement, Bonferroni t-test) and post-test occurrence of SWRs (p < 0.05 versus all measurements except first bin and post-learning bin, Bonferroni t-test). These findings reveal that a single learning session is sufficient to produce an increase in the hippocampal SWR occurrence rate, thereby reflecting an important involvement of hippocampal oscillations in memory formation. However, contrasting with memory paradigms involving multiple training sessions^5^, no significant changes in normalized power, duration or frequency of SWRs were observed either after encoding or recognition testing of our vehicle- and Aβo -injected mice (F < 1, NS for all comparisons, n = 7, data not shown).

In sharp contrast, cognitively-challenged Aβ mice exhibited impaired SWR patterns compared to PBS-control mice. Namely, the encoding and recognition-induced peaks in the SWR occurrence rate observed in the control group (Fig. 5b, upper panel) were abolished in Aβ animals (Fig. 5c, upper panel). Indeed, ANOVA with “time bins” as repeated measurements and “treatment” (Aβ vs PBS) as between-subjects variable showed significant effect of “time bins” (F_12,9_ = 24.02, p < 0.0001) as well as “time bins” × “treatment” interaction (F_12,9_ = 3.12, p = 0.002), indicating that the dynamic of SWRs during the course of experiment was different in the two groups of animals. Finally, comparison of occurrence of SWRs during all time bins between control and Aβo-injected animals revealed significant difference for the post-encoding period (t_12_ = 2.48, p = 0.029) and a difference close to significance for the post-test period (t_12_ = 2.09, p = 0.058) with all other measurements remaining similar between the two groups (p > 0.2).

When refining our analysis of SWR dynamics by restricting it to shorter time bins of 20 min, we found similar patterns of SWR occurrence in PBS-controls and Aβo-injected mice (Fig. 5b,c, bottom panels). Since accuracy of ripple rate estimation decreases for very short SWS bouts, we only took into account animals which expressed at least a 5 cumulated min of SWS in the bin. This resulted in unequal animal numbers in the 20 min bins (see numbers within bars, bottom panels of Fig. 5) and rendered impossible the use of an ANOVA similar to that performed for 40 min bins histograms. However, a comparison of SWR occurrence rate during 20 min bins confirmed a significant difference between vehicle- and Aβo-injected groups during the second post-encoding bin (t_12_ = 2.43, p = 0.032, n = 7).

**Figure 6:**
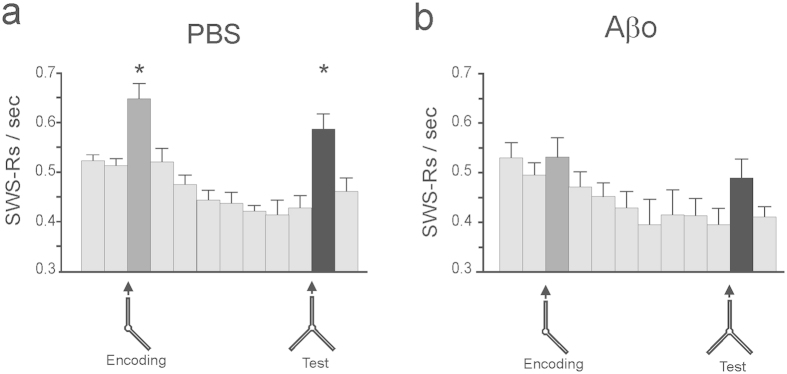
Time course of SWRs occurrence rate in 15 min bins of SWS. This restrictive analysis enabled to control for the differential amount of SWS per time bin among recorded mice and revealed the same pattern of effects as depicted in Fig. 5. Encoding- and recognition-induced peaks of SWR occurrence are present in the PBS-control group (**a**), *p < 0.01 versus other measurement, Bonferroni t-test, n = 7) but abolished in the Aβ group (**b**), NS vs all other measurements, Bonferroni t-test, n = 7).

Binning total time and not SWS time might imply that for some animals the rate of SWRs for the first 40 min bin was calculated for instance over the first 5 min of SWS whereas for another time bin, it was calculated over the first 30 min, depending on how much the animal has slept during this time. Therefore we performed additional analysis taking into account time bins corresponding to SWS only (Fig. 6). For each animal, the duration of SWS episodes was cumulated from three distinct parts of the behavioral experiment: 1) before encoding, 2) between encoding and test and 3) after test. Thus, duration of SWR episodes was divided into 15 min bins within each part and the SWR occurrence rate was expressed as the number of ripples occurring within each 15 min SWS bin (Fig. 6). This analysis confirmed the abolishment of learning-induced increase of SWRs occurrence rate in Aβo-injected animals. Indeed, besides the main effect of repetition (“SWS bins”) (F_12,11_ = 22.56, p < 0.0001) a two-way ANOVA showed significant “SWS bins” × “treatment” interaction (F_12,11_ = 2.33, p = 0.012), indicating a different time course of SWRs occurrence in the two groups. Moreover, a one-way ANOVA showed a significant main effect of repetition in the PBS-control group (F_6,11_ = 15.1, p < 0.0001) and the Aβo-injected group (F_6,11_ = 9.11, p < 0.0001). In the control group the post-hoc analysis revealed a significant increase of post-learning and post-test occurrence of SWRs (p < 0.05 versus all other measurements, Bonferroni t-test). By contrast, in Aβ animals no difference between occurrence rate in SWS bins was found (NS for all comparisons, Bonferroni t-test). Also direct comparisons of the SWRs occurrence rates in all SWS bins between the two groups showed significant difference for the post-encoding period (t_12_ = 2.41, p = 0.032), a difference approaching significance for the post-test period (t_12_ = 2.0, p = 0.068) and no difference for all remaining bins (p > 0.2).

Rather than being memory-specific, the testing-induced increase in the occurrence of SWRs could be the consequence of homeostasis maintenance of the neuronal circuits underlying a sustained period of exploration in the Y-maze. To control for this potential confound, we analyzed in detail the profile of exploration of experimental Aβo- and vehicle-injected mice used for hippocampal recordings during both encoding and test phases in the Y-maze. Distance traveled (Encoding: PBS mice, 3514.5 ± 134.1 cm; Aβ mice, 3107.1 ± 248.9 cm; Test: PBS mice, 1465 ± 496.7 cm; Aβ mice, 1576.5 ± 126.8 cm), speed (Encoding: PBS mice, 9.2 ± 0.3 cm/s; Aβ mice, 9.1 ± 0.1 cm/s; Test: PBS mice, 9 ± 0.5 cm/s; Aβ mice, 8.7 ± 0.3 cm/s) and percentage of immobility (Encoding: PBS mice, 35 ± 2.3%; Aβ mice, 37.1 ± 2.8%; Test: PBS mice, 42.6 ± 19.1%; Aβ mice, 36.4 ± 5.3%) were similar across the two groups. The fact that vehicle- and Aβo–injected mice underwent the exact same procedure coupled to the observation that these mice explored as well as encoded similarly (as shown by a similar between-group recognition performance at the 10 min delay) enables to exclude a nonspecific contribution of homeostasis maintenance to the observed memory-induced changes in SWR occurrence. We did not observe any correlation between SWR occurrence and recognition memory performance in the Y-maze (data not shown), possibly because exploration time in new arm as the main readout of recognition performance can only be measured over one single trial (innate test with no repeated observations) and does not fully capture the vividness of the memory.

Together, these results demonstrate that the deleterious effect of Aβos on the dynamics of SWRs is activity-dependent in nature and only effective in cognitively-demanding situations requiring hippocampal processing.

## Discussion

Recognition memory, a subdivision of episodic memory, is of particular interest in the context of AD as this form of memory is typically affected during the early stages of this neurodegenerative disease^17^. We adapted the classical two-trial recognition procedure in the Y-maze to the 8-arm radial maze in order to promote reliance on distal cues, thereby enhancing the spatial cognitive demand of the testing procedure. This adaptation highlighted the potential for a long-lasting spatial recognition memory which could last for at least 24 hours. Its hippocampal-dependent nature was confirmed by region-specific post-encoding inactivation of the hippocampus which impaired performance, thus pointing to the functional involvement of this brain region in supporting the formation and expression of spatial recognition memory.

Consistent with previous findings, our study reveals a transitory increase in the occurrence rate of hippocampal SWRs following a spatial learning episode, further strengthening the functional implication of SWRs in the progressive stabilization of spatial information during the course of memory consolidation processes^7^. We identified two peaks in hippocampal SWR occurrence during the 40 min following either the encoding or the recognition phases, a neuronal signature similar to that reported in associative spatial memory tasks in the rat^5^,^18^. However, contrasting with an increase in ripple magnitude after new associative learning or long-term memory retrieval^5^, we did not find any changes of SWR duration or normalized power. This differential pattern may be due to the fact that in our recognition memory paradigm, mice were exposed only once to the maze prior to engaging into SWS when ripples were recorded whereas in the previous work, animals were subjected to intensive multiple training sessions in which they had to extract specific learning rules. Moreover, our testing procedure relied on the innate preference of rodents for novelty and did not involve any reward-associated learning. Noteworthy is the transitory pattern of the two hippocampal SWR occurrence peaks observed upon encoding and recognition testing. They lasted only 40 minutes, a temporal dynamics which suggests that they may have acted primarily as a triggering switch during SWS for subsequent long-lasting cellular and molecular changes in weight and wiring plasticity within hippocampal cell assemblies actively engaged in processing the spatial layout of the maze environment. Thus, post-encoding SWRs could be predominantly involved in spatial memory formation and have a growing importance in its stabilization as memory mature over time. Accordingly, SWRs would not be required for expression of memory shortly after encoding (no impairment is seen at 10 min) but would be required to initiate stabilization processes and subsequent access to the memory trace upon retrieval over longer time points. This could explain why, at 2 h, a memory impairment starts to emerge (although not significant) and becomes more prevalent at 4 h, possibly because the lack of SWR peak following encoding in Aβo-injected mice resulted in a failure in triggering the adequate progressive stabilization processes during SWS of the general spatial configuration of the maze. Another proposition regarding the transitory nature of the two hippocampal SWR occurrence peaks, although speculative, is that in a more ethological situation wherein animals have to process and potentially remember an array of successive information, it will be more advantageous that these pieces of information are processed as quickly as possible (i.e. short peak of SWRs) to avoid overlap of hippocampal replays during subsequent periods of quiet wakefulness or phases of sleep. Furthermore, the ability of the animal to later recognize the maze environment requires the successful reinstatement of previously stabilized hippocampal place maps. SWRs are likely candidates for such a process of stabilization by strengthening spatial cell assemblies^19^. Functionally, the encoding- and recognition-induced SWR drives, we identified, may convey different roles. Upon encoding, the hippocampal SWR occurrence peak could initiate the progressive stabilization during SWS of the general spatial configuration of the maze (i.e. access to two arms of the maze). Upon recognition testing, the SWR drive may reflect the partial remapping of hippocampal place fields related to the formation of an updated representation of the environment in which one additional arm of the maze is now available.

Because SWRs are triggered in cognitively challenged animals, their dysfunctional patterns are expected to impair memory-related processes. Accordingly, when disrupted experimentally, abnormal SWR signatures leads to impaired spatial learning^6^,^20^,^21^. With regards to neurodegenerative diseases such as AD, the functional contribution of SWRs to the reported impairments in spatial memory remains however poorly understood. To implement the observation that cognitive deficits of AD patients are correlated to soluble Aβ levels rather than plaque-development *per se*^22^, we chose to inject intracerebroventrically synthetic forms of Aβos in mice. This model produces cognitive deficits much faster than other transgenic animal models in which memory impairments develop only within months and enables rigorous control over the time course of AD symptomatology^23^. We found that Aβo-injected mice exhibited faster forgetting compared to controls, a memory profile pointing to an inability to form and stabilize, or retrieve, long-lasting memories. Because the spatial recognition procedure relies on the natural tendency of animals to seek novelty, the possibility remains that the Aβo-treatment impacted other non-mnesic behavioral components, such as for instance reduced attraction to the novel arm or novelty-related increased in anxiety that would prevent exploration of the novel arm during the testing phase despite remembering the previously explored arms. However, the observation of an intact recognition memory at a very short delay (10 minutes) in Aβo-injected mice makes these potential confounding factors unlikely. It further strengthens the existence of altered memory consolidation and retrieval processes, two mechanistic accounts already suggested in other transgenic models of AD in which only early state of Aβ aggregation is present without plaque formation^16^.

We found that the accelerated memory decay of Aβ-treated mice was associated with an abolishment of the two time-limited peaks of SWRs normally seen in controls. The fact that vehicle- and Aβo–injected mice underwent the exact same procedure coupled to the observation that these mice explored as well as encoded similarly (as shown by a similar between-group recognition performance at the 10 min delay) enables to minimize the involvement of nonspecific aspects of our testing procedure. Although we did not record hippocampal activity of mice during testing in the Y-maze, it is likely that mice of both groups maintained a similar theta brain state during exploration of the maze. Thus, the increase in SWR occurrence observed after both encoding and testing phases in vehicle-injected, but not Aβo-injected mice, is likely to be predominantly related to the memory component of the testing procedure and not to a differential requirement of neuronal homeostasis between the two tested groups. Altogether our data suggest that the two time-limited peaks of SWRs likely constitute a prerequisite for the formation and accurate expression of spatial recognition memory.

At the mechanistic level, memory reactivation is considered as the core iterative mechanism in contemporary consolidation models. Hippocampal place cells that were co-active during spatial exploration exhibit correlated firing patterns during SWS, revealing a replay mechanism. Importantly, hippocampal replay retains the original temporal order, and occurs preferentially during the occurrence of SWRs^7^,^9^,^19^, thus conferring to these specific offline oscillations a privileged role in promoting weight and wiring synaptic plasticity and in coordinating memory consolidation across hippocampal-cortical networks. Importantly, our results demonstrate for the first time that it is a lack of post-learning increase in the SWR occurrence rate, and not an absolute absence of SWRs (still generated normally in Aβ mice prior to memory testing), which may be responsible for the impaired memory profile of Aβ mice. This suggests no alteration of the neuronal mechanism underlying the generation of SWRs but points instead to its inability to respond adequately to a specific cognitive demand. This statement is further supported by a complete preservation of SWR properties in Aβ-treated mice in resting conditions, a finding which is also in agreement with the unaffected ongoing SWR activity demonstrated in slices from transgenic AD mice^13^ and rat Aβ-treated slices^24^. Interestingly, the properties of SWRs are altered only when neurofibrillary tangles and neurodegeneration are detected, two hallmarks of AD which appear during later stages of the AD pathology^25^. This finding highlights another mechanism triggered by the AD pathology which can affect SWR properties over a different time course.

Although many cellular and synaptic mechanisms can explain the Aβ-induced lack of SWRs triggered upon a cognitive challenge, one putative candidate is NMDAR-induced synaptic plasticity. Indeed, it has been demonstrated that high level of Aβos can alter glutamatergic synaptic transmission which in turn can lead to synaptic loss^26^. Moreover, the post-learning increase of SWR occurrence has been recently proposed to result from NMDA receptor plasticity and early (upon encoding) neuronal tagging of hippocampal-cortical networks, a NMDAR-dependent neurobiological process required for the progressive embedding of memory traces within hippocampal-cortical networks during sleep and resting periods^21^,^27^. It is therefore possible that the early Aβ-induced alteration of NMDA receptor function may preclude the dynamic response of hippocampal networks to post-learning requirement.

In conclusion, our data provide novel insights into the functional involvement of SWRs in the spatial memory impairments observed in AD. While unaffected in basal conditions, the occurrence patterns of hippocampal SWRs associated with either encoding or expression of recognition memory were specifically disrupted in the event of a challenging situation. Because Aβ-treated mice were able to form short-term but not long-term recognition memory, the absence of the SWR occurrence peak following encoding likely impacted predominantly consolidation processes involved in the subsequent stabilization of the hippocampal memory trace and not encoding processes *per se*. The failure in expressing long-term recognition memory of Aβ mice was also associated with a lack of a dedicated SWR occurrence peak, possibly indicating that the memory has not been properly stabilized (faster forgetting) or that access to a partially degraded trace was no longer possible. While highlighting the crucial roles played by SWRs dynamics in hippocampal memory processing, our findings also identified the absence of learning-induced SWR occurrence rates as a potentially early marker of AD.

## Materials and Methods

### Preparation of Aβos

The Aβ_(1–42)_ peptide was obtained from NeuraTest (Bordeaux, France). Prior to resuspension, each vial was allowed to equilibrate to room temperature for 30 min to avoid condensation upon opening the vial. The first step in the resuspension of the lyophilized peptide was treatment in 1,1,1,3,3,3-hexafluoro-2-propanol (Sigma-Aldrich, L’Isle d’Abeau, France). Each vial of peptide was diluted in 100% HFIP to 1 mM. The clear solution containing the dissolved peptide was then aliquoted in microcentrifuge tubes. The HFIP was evaporated using a gentle stream of nitrogen gas under the fume hood. Immediately prior to use, the HFIP-treated aliquots were carefully and completely resuspended to 2 mM in anhydrous dimethyl sulfoxide (Sigma-Aldrich, L’Isle d’Abeau, France) by pipette mixing followed by bath sonication for 15 min. Then, the sample was dissolved in 95 μl of ice-cold PBS, immediately vortexed for 30 s, and incubated at 4 °C for 24 h. Final concentration obtained was 100 μM (store at −80 °C). This Aβ preparation has been characterized previously in Stine *et al.*^14^ and validated *in vivo* in Balducci *et al.*^28^.

### Immunoblotting

Electrophoresis was performed on 4–12% NuPAGE Bis-Tris polyacrylamide gels (Invitrogen, France). After size separation within the gel, proteins were transferred to a polyvinylidenedifluoride (PVDF) membrane (Polyscreen® membrane, Perkin Elmer, France). Membranes were blocked with a solution containing 0.1% Tween 20 and 200 mM Tris buffered solution (TTBS) complemented with 5% non-fat dry milk during 30 min and incubated with mouse β amyloid 1–16 (6E10; Eurogentec, France) monoclonal antibody at 4 °C overnight under gentle agitation. Incubation with the secondary fluorescent-conjugated antibody was performed during 1 h at room temperature. After 3 washes with TTBS and one with PBS, the membrane was scanned using a Licor Aerius automated infrared imaging system according to manufacturer’s instructions.

### Animals and surgery

After habituation to the vivarium conditions, 83 male C57BL/6J mice (3–4 months) underwent stereotaxic surgery under deep isoflurane anesthesia. As a model of AD^23^, we have used an intracerebroventricular injection of Aβo as previously described by Balducci *et al.*^28^. One injection cannula connected via a catheter to a 5 μl Hamilton syringe was aimed at the right lateral ventricle using the following coordinates: anteroposterior (AP) relative to bregma, −1.0 mm; lateral (L) to midline, 1.3 mm; ventral (V) from the skull surface, −2.0 mm. A 4 μl solution of Aβos or phosphate buffered saline (PBS) containing 5% of DMSO used as vehicle was infused at a rate of 0.5 μl/min with an injection pump controlling the syringe (Harvard Apparatus, Holliston, MA, USA). Bilateral electrodes consisting of an insulated tungsten wire (diameter 35 μm, California Fine Wires) were implanted into the CA1 region of the hippocampus (AP: −2.0 mm, L: ±1.5 mm, V: −1.05 mm). Reference and ground electrodes were implanted into the cerebellum. The electromyogram (EMG) electrode was inserted into the neck muscles. All electrodes were welded to a 6-pin connector attached to the skull with dental acrylic cement. For mice infused with lidocaine (4% in artificial cerebrospinal fluid (aCSF), Sigma-Aldrich), bilateral guide cannulas were implanted into the dorsal hippocampus as previously described^29^. Lidocaine (0.5 μl per side) was bilaterally delivered by means of cannulas connected to a 5 μl syringe mounted on a perfusion pump. Experimental procedures complied with official European Guidelines for the care and use of laboratory animals (directive 2010/63/UE) and were approved by the ethical committee of the University of Bordeaux (protocol A50120159).

### Spatial recognition memory

The Y-maze two-trial procedure is routinely used to examine spatial recognition memory and takes advantage of the innate tendency of rodents to explore novel environments^30^. To increase its spatial cognitive demand, we adapted it to the 8-arm radial maze (Imetronic, France) in which only three arms were used to form a Y-shape (90°-135°-135° between the arms). Each arm was 62 cm long and 12 cm wide and radiated from a central platform (32 cm in diameter). Behavioral procedure was composed of the exploration (encoding) phase and the recognition phase, which were separated by various inter-trial intervals (ITIs). During the encoding trial, one of the three available arms was closed. The mouse was positioned on the central platform of the maze and allowed to explore the two available arms during 10 min. During the recognition trial, the animal could explore all three arms during a 5 min period. The time spent by the animal in each of the three arms of the maze was automatically recorded during the encoding and the test phase, with the entrance into an arm being scored when the first half of the animal’s body was inside that arm. Thus, the total time spent in all three arms corresponded to the total exploration time. Mice normally tend to explore the previously blocked arm (novel arm) of the maze more often than the previously accessible (familiar) ones. Because this behavioral paradigm relies on novelty seeking, the recognition trial should not be repeated and animals were used only once. Discriminating the novel arm from the two familiar arms is thus considered as an index of spatial recognition memory. Memory performance was expressed as the percentage of time spent in novel arm calculated as follows: (time spent in novel arm/time spent in all three arms) × 100. The time spent on the central platform of the maze was excluded from the calculation of performance. Chance level was set at 33% of the exploration time. A detailed profile of exploration of the maze by each animal during encoding and test phases (distance traveled in each arm including central platform, speed of exploration and % of immobility provided by the Imetronic videotracking system coupled to the maze) was also generated to examine the patterns of exploration of vehicle- and Aβo-injected groups.

### Electrophysiological recordings and data processing

Daily recordings were performed from 9 am to 4 pm in a closed opaque and dimly illuminated box which could host the animal’s home cage. The mouse head connector was linked to amplifiers by a soft cable allowing free motions of the animal. Behavior was tracked with a video camera. Electroencephalograms and EMG signals were amplified by a differential home-made AC amplifier, digitized at 32 kHz with 16-bit resolution using CED Power 1401 converter and Spike2 software (Cambridge Electronic Design), and stored on a PC for off-line analysis. To obtain local field potential (LFP) signals, raw signals were first processed by NDManager^31^ which provided both filtering and down-sampling (from 32 kHz to 1250 Hz) and thereafter filtered using Chebyshev Type II filter (order 4) in the 100–250 Hz band. Sonic Vizualizer was used to display and analyze the spectrograms. EMG was band-pass filtered to 250–350 Hz. Power spectra of delta (1–5 Hz) and theta (5–10 Hz) frequency band were calculated continuously. Brain states corresponding to awake, REM and slow wave sleep (SWS) states were manually scored by experimenter using EMG, spectrogram delta/theta ratios as cues as well as video-recording. SWS states were identified as episodes of immobility (tonic EMG) and high delta power. SWS bouts separated by less than 3 seconds were merged. REM states were identified as episodes of high theta and low delta power accompanied by atonic neck EMG recording. After filtering of LFP signals in the 100–250 Hz band, SWRs were detected using the normalized squared signal (NSS)(FMA Toolbox http://fmatoolbox.sourceforge.net/API/FMAToolbox/Analyses/FindRipples.html) only during periods classified as SWS. SWRs were identified by thresholding the NSS if its envelope exceeded 2 SD and peak exceeded 5 SD. The time points when the NSS crossed 2 SD were considered as onset and offset of a SWR. Episodes lasting longer than 100 ms were excluded from the analysis whereas episodes separated by less than 30 ms were merged. The occurrence rate was expressed as number of SWRs per sec of SWS (SWS-Rs/sec). Normalized power was calculated as maximum of NSS within a ripple. Time bins containing less than 5 min of a total duration of SWS were excluded from analysis of SWRs dynamics.

### Quantification of Aβ_(1–42)_ peptide

Twenty-four hours (n = 4) and 15 days (n = 4) after the intracerebroventricular injection of Aβ_(1–42)_ oligomers, mice were deeply anesthetized with isoflurane 5% and right hippocampi were carefully harvested and homogenized in lysis buffer containing 20 mM HEPES, 0.15 mM NaCl, 1% triton ×100, 1% deoxycholic acid, 1% SDS, pH 7.5 and supplemented with a protease inhibitor cocktail (Sigma-Aldrich, L’Isle d’Abeau, France). Protein amounts of hippocampal homogenates were determined by the Bradford’s protein assay and normalized to 500 μg of protein per sample. Hippocampal concentration of Aβ_(1–42)_ peptide was evaluated by ELISA (Human Amyloid beta 42 Ultrasensitive ELISA Kit, Thermofisher, France). This kit specifically detects soluble forms of human Aβ_(1–42)_ peptides with negligible cross-reactivity to human Aβ_(1–40)_ or mouse Aβ_(1–42)_ forms. Aβ concentration in samples was determined by comparison to a standard curve (0–250 pg/ml). The absorbance at 450 nm was read using a microplate reader.

### Statistical analyzes

Results were expressed as mean ± SEM. After checking normality of distributions with the Shapiro-Wilk test as well as homogeneity of variance using the Levene’s test, data analyses were performed using analyses of variance (ANOVAs) followed by post-hoc comparisons performed by t-test with Bonferroni correction where appropriate. For ANOVAs with repeated measures, we additionally tested sphericity by means of the Mauchly’s test. Values of p < 0.05 were considered as significant.

## Additional Information

**How to cite this article**: Nicole, O. *et al.* Soluble amyloid beta oligomers block the learning-induced increase in hippocampal sharp wave-ripple rate and impair spatial memory formation. *Sci. Rep.* **6**, 22728; doi: 10.1038/srep22728 (2016).
